# Adverse Effects Associated with Protein Intake above the Recommended Dietary Allowance for Adults

**DOI:** 10.5402/2013/126929

**Published:** 2013-07-18

**Authors:** Ioannis Delimaris

**Affiliations:** External Postdoctoral Research Team, Biology Unit, Faculty of Human Sciences, University of Thessaly, 38221 Volos, Greece

## Abstract

*Background*. While high-protein consumption—above the current recommended dietary allowance for adults (RDA: 0.8 g protein/kg body weight/day)—is increasing in popularity, there is a lack of data on its potential adverse effects. *Objective*. To determine the potential disease risks due to high protein/high meat intake obtained from diet and/or nutritional supplements in humans. *Design*. Review. *Subjects*. Healthy adult male and female subjects. *Method*. In order to identify relevant studies, the electronic databases, Medline and Google Scholar, were searched using the terms:“high protein diet,” “protein overconsumption,” “protein overuse,” and “high meat diet.” Papers not in English were excluded. Further studies were identified by citations in retrieved papers. *Results*. 32 studies (21 experimental human studies and 11 reviews) were identified. The adverse effects associated with long-term high protein/high meat intake in humans were (a) disorders of bone and calcium homeostasis, (b) disorders of renal function, (c) increased cancer risk, (d) disorders of liver function, and (e) precipitated progression of coronary artery disease. *Conclusions*. The findings of the present study suggest that there is currently no reasonable scientific basis in the literature to recommend protein consumption above the current RDA (high protein diet) for healthy adults due to its potential disease risks. Further research needs to be carried out in this area, including large randomized controlled trials.

## 1. Introduction

Protein is an essential macronutrient needed by the human body for growth and maintenance. Foods rich in animal protein are meat, fish, eggs, poultry, and dairy products, while plant foods high in protein are mainly legumes, nuts, and grains. The current recommended dietary allowance (RDA) for protein is 0.8 g protein/kg body weight/day for adults (for children 1.5 g protein/kg body weight/day, and for adolescents 1.0 g protein/kg body weight/day) [1]. However, high protein diets (defined as an intake above the current RDA) are promoted intensively by the nutritional supplements industry and they are considered to be “the gold standard” by many athletes (especially bodybuilders) for muscle development and/or body fat loss. On the other hand, several scientists claim that the overuse of protein supplements or high dietary protein intake could cause disorders to human health [1–7]. The aim of this review study is to determine the potential health dangers due to high protein/high meat intake obtained from diet or nutritional supplements based on the human studies existent in the literature. During the period of October 2012–May 2013, a search was carried out in the databases PubMed (1967 to present) and Google Scholar (1966 to present). There were included studies in English language which had analyzed the potential health dangers due to long-term high protein intake obtained from diet or nutritional supplements in humans. The titles and the abstracts of the initial studies identified were searched in order to determine if they satisfy the selection criteria. The integral text of selected titles was extracted and the reference list of selected articles was consulted in order to find out other relevant publications. 32 studies (21 experimental human studies and 11 reviews) were identified which comprised data related to the potential adverse effects of protein overconsumption using the following search terms: “high protein diet,” “protein overconsumption,” “protein overuse,” and “high meat diet.” The experimental human studies' features included in the study are conveyed in Table 1 (reviews are not included).

## 2. Disorders of Bone and Calcium Homeostasis

Diet which is high in protein generates a large amount of acid in body fluids [2]. The kidneys respond to this dietary acid challenge with net acid excretion, and, concurrently, the skeleton supplies buffer by active resorption of bone resulting in excessive calcium loss [2]. Moreover, acid loading directly inhibits renal calcium reabsorption leading to hypercalciuria in combination with the exorbitant bone loss [3, 4]. In a metabolic study an increase in protein intake from about 47 to 112 g caused an increase in urinary calcium and a decrease in calcium retention. The data indicated that protein-induced hypercalciuria was due to an elevation in glomerular filtration rate and a lower fractional renal tubular reabsorption of calcium, the latter of which caused by the increased acid load on the renal tubular cells [8]. Another study on subjects consuming diets containing 48 g protein daily to 142 g showed that urinary calcium doubled, while the calcium balance became negative [9]. In addition, the effect of dietary protein on markers of bone turnover has been evaluated [10]. In this study the subjects were on a well-balanced diet for 2 weeks which was followed by 4 days of an experimental diet containing one of three levels of protein (low, medium, or high). Urinary calcium excretion was significantly higher, and urinary N-telopeptide excretion (indicator of bone resorption) was significantly greater during the high protein than during the low protein intake. Data suggested that, at high levels of dietary protein, at least a portion of the increase in urinary calcium reflected increased bone resorption [10]. Additionally, subjects on a low-carbohydrate high-protein (LCHP) diet for 6 weeks had increased urinary calcium levels, decreased calcium balance, and decreased serum osteocalcin concentrations [11]. In a prospective study, protein was associated with an increased risk of forearm fracture for women who consumed more than 95 g per day compared with those who consumed less than 68 g per day. Women who consumed five or more servings of red meat per week also had a significantly increased risk of forearm fracture compared with women who ate red meat less than once per week [12]. Furthermore, the effect of high-protein diets on the excretion of calcium in urine was evaluated in normal persons and patients with nephrolithiasis. All subjects were given diets containing 0.5 g protein/kg/day, while, during the experimental phase, each person received an additional 1.5 g protein/kg/day. There was a consistent increase in urinary calcium with the high-protein diet averaging 88% above control in the normals and 82% in the patients [13]. Moreover, in a study where protein intake was varied from 47 g/day (low protein diet) to 95 g/day (medium protein diet) and to 142 g/day (high protein diet) the urinary calcium increased significantly with each increase in protein (168, 240, and 301 mg, resp.) [14]. In addition, it has been shown that increasing the protein intake from 48 to 141 g daily caused a highly significant elevation in urinary calcium, the mean daily values being 175 and 338 mg, respectively [15]. In another study the relationship of animal protein rich diet to calcium metabolism was investigated during a 12-day dietary period. An increase in urinary calcium excretion was found indicating that the animal protein-induced calciuric response could be a risk factor for the development of osteoporosis [16]. Notably, it has been shown that the consumption of high calcium diets is unlikely to prevent the negative calcium balance and probable bone loss induced by the consumption of high protein diets (protein-induced hypercalciuria) [17]. In this experiment (a 95-day metabolic study) subjects received formula diets supplying 12 g nitrogen or 36 g nitrogen, and approximately 1400 mg calcium per day. Overall calcium balance was −37 mg/day on the 12 g nitrogen diet, and significantly lower at −137 mg/day in subjects consuming the high protein diet [17]. Additionally, dietary excess (2 g/kg/day) in animal protein for 1 week led to significant changes in urinary calcium excretion rates [18]. Furthermore, in an interesting study the effects on urinary calcium levels of increasing dietary protein from 50 to 150 g protein were compared with those of increasing the sulfur amino acids to simulate the amounts present in the 150 g protein diet. The increase in protein intake caused urinary calcium to double, while sulfur amino acids added to the low protein diet also caused urinary calcium to increase [19]. Moreover, a prospective cohort study showed that a high ratio of dietary animal to vegetable protein increases the rate of bone loss and the risk of fracture in postmenopausal women. Animal foods provide predominantly acid precursors, whereas protein in vegetable foods is accompanied by base precursors not found in animal foods. Imbalance between dietary acid and base precursors leads to a chronic net dietary acid load that may have adverse consequences on bone. An increase in vegetable protein intake and a decrease in animal protein intake may decrease bone loss and the risk of hip fracture [20].

## 3. Disorders of Renal Function

**Figure 1:**
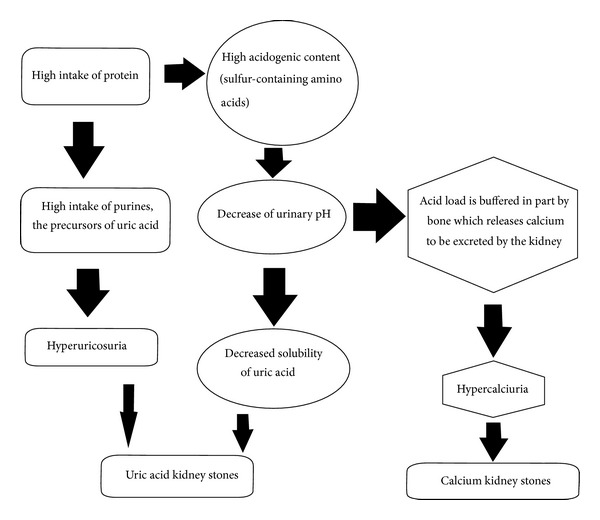
Pathobiochemical mechanisms of animal protein-induced nephrolithiasis.

Low fluid intake and excessive intake of protein are important risk factors for kidney stones [3]. Protein ingestion increases renal acid excretion, and acid loads, in turn, may be buffered in part by bone, which releases calcium to be excreted by the kidney. This protein-induced hypercalciuria could lead to the formation of calcium kidney stones [4]. Furthermore, animal protein is also the major dietary source of purines, the precursors of uric acid. Excessive intake of animal protein is therefore associated with hyperuricosuria, a condition present in some uric acid stone formers [5]. Uric acid solubility is largely determined by the urinary pH. As the pH falls below 5.5 to 6.0, the solubility of uric acid decreases, and uric acid precipitates, even if hyperuricosuria is not present [5]. The pathobiochemical mechanisms of animal protein-induced nephrolithiasis are shown in Figure 1. An interesting study on the effects of protein overload on stone-forming propensity showed that consumption of high-protein diet for 6 weeks delivers a marked acid load to the kidney and increases the risk for stone formation (urinary citrate levels decreased, and urinary saturation of undissociated uric acid increased) [11]. Furthermore, in a study of three 12-day dietary periods during which the diet of the subjects contained vegetable protein, vegetable and egg protein, or animal protein, it was found that the animal protein-rich diet was associated with the highest excretion of undissociated uric acid due to the reduction in urinary pH [16]. Moreover, citrate excretion was reduced because of the acid load, and urinary crystallization studies revealed that the animal protein diet conferred an increased risk for uric acid stones [16]. In another study it was shown that a high protein intake induced changes in urinary uric acid and citrate excretion rates and a decrease in the ability of urines to inhibit calcium oxalate monohydrate crystal agglomeration [18]. The decreased ability of urines to inhibit the agglomeration of calcium oxalate crystals could provide a possible physicochemical explanation for the adverse effects of high-protein diet on renal stone formation [18]. Additionally, it has been indicated that high-protein intake could cause increased glomerular filtration rate and decreased fractional renal tubular reabsorption of calcium and urinary sodium [19]. In another study, healthy subjects with a history of renal stones fed on a low (LPD) and a high (HPD) animal protein diet; after 2 weeks it was found that high dietary intake of purine-rich animal protein had an impact on urinary urate excretion and supersaturation in renal stone disease [21]. There was an increase in urinary urate, urinary acid excretion, ammonium ion excretion, and uric acid supersaturation and a fall in urine pH on HPD. The risk of forming uric acid or ammonium urate crystals or stones in the urine was increased on a high protein diet [21]. Moreover, in a prospective cohort study it was investigated whether protein intake influences the rate of renal function change over an 11-year period. The results showed that high total protein intake, particularly high intake of nondairy animal protein, may accelerate renal function decline in women with mild renal insufficiency [22]. Furthermore, a study about the short-term effect of increasing the dietary consumption of animal protein on the urinary risk factors for stone-formation showed increased levels of urinary calcium and oxalate. The accompanying increase in dietary purine caused an increase in the excretion of uric acid. The overall relative probability of forming stones, calculated from a combination of the risk factors, was markedly increased (250%) throughout the period of high animal protein ingestion [23].

## 4. Increased Cancer Risk, Disorders of Liver Function, and Precipitated Progression of Coronary Artery Disease

Up to 80% of breast, bowel, and prostate cancers are attributed to dietary practices, and international comparisons show positive associations with high meat diet [6]. The association, however, seems to have been more consistently found for red meat or processed meat and colorectal cancer [7]. Possible mechanisms include the formation of heterocyclic amines in meat when it is cooked. These heterocyclic amines require acetylation by P450 enzymes, and individuals with the fast-acetylating genotype who eat high amounts of meat may be at increased risk of large-bowel cancer [6]. It should be noticed that red meat is the main dietary source of saturated fat, which has been associated with breast and colorectal cancers [1]. Moreover, NH_3_ and N-nitroso compounds (NOC) formed from residues by bacteria in the large bowel are probably also important. NH_3_ is a promotor of large-bowel tumours chemically induced by NOC, and some of the chromosomal mutations found in human colorectal cancer are consistent with effects of NOC and heterocyclic amines [6]. In a cohort study subjects who were free of diagnosed cancer completed a validated food frequency questionnaire and provided detailed information on other lifestyle and health-related factors. An elevated risk of colon cancer was associated with red meat intake [24]. Men who ate beef, pork, or lamb as a main dish five or more times per week had an elevated relative risk compared to men eating these foods less than once per month. The association with red meat was not confounded appreciably by other dietary factors, physical activity, body mass, alcohol intake, cigarette smoking, or aspirin use [24]. Furthermore, in a prospective study subjects without a history of cancer, inflammatory bowel disease, or familial polyposis completed a dietary questionnaire [25]. After adjustment for total energy intake, animal fat was positively associated with the risk of colon cancer. The relative risk of colon cancer in subjects who ate beef, pork, or lamb as a main dish every day was increased, as compared with those reporting consumption less than once a month [25]. In an interesting study the overall data set derived from an integrated series of case-control studies included histologically confirmed neoplasms; controls were patients admitted to hospital for acute, nonneoplastic conditions unrelated to long-term modifications in diet [26]. The multivariate odds ratios (ORs) for the highest tertile of red meat intake (≥7 times/week) compared with the lowest (≤3 times/week) were 1.6 for stomach, 1.9 for colon, 1.7 for rectal, 1.6 for pancreatic, 1.6 for bladder, 1.2 for breast, 1.5 for endometrial, and 1.3 for ovarian cancers. Thus, reducing red meat intake might lower the risk for several common neoplasms [26]. Moreover, highprotein/high meat diet could cause disorders of liver function and precipitated progression of coronary artery disease. Hyperalbuminemia and elevated transaminases have been associated with high-protein diet [27]. Individuals on high protein supplements developed intermittent abdominal pain, transient elevations in transaminases, and hyperalbuminemia without there being any identifiable cause. The symptoms and abnormalities on the laboratory tests resolved after the high protein intake was discontinued [27]. In a case-control study, subjects (treatment group/TG) were studied for 1 year by using myocardial perfusion imaging (MPI), echocardiography (ECHO), and serial blood work [28]. MPI and ECHO were performed at the beginning and end of the study for each individual. The TG group studied modified their dietary intake as instructed. Additional subjects (high protein group/HPG) elected a different dietary regimen consisting of a “high-protein” diet [28]. Subjects in the TG demonstrated a reduction in each of the independent variables studied with regression in both the extent and severity of coronary artery disease (CAD) as quantitatively measured by MPI. Individuals in the HPG showed worsening of their independent variables. These results would suggest that high-protein diets may precipitate progression of CAD through increases in lipid deposition and inflammatory and coagulation pathways [28].

## 5. Conclusions

Despite the fact that short-term high protein diet could be necessary in several pathological conditions (malnutrition, sarcopenia, etc.), it is evident that “too much of a good thing” in diet could be useless or even harmful for healthy individuals [1, 29]. Many adults or even adolescents (especially athletes or body builders) self-prescribe protein supplements and overlook the risks of using them, mainly due to misguided beliefs in their performance-enhancing abilities [30]. Individuals who follow these diets are therefore at risk [31]. Extra protein is not used efficiently by the body and may impose a metabolic burden on the bones, kidneys, and liver. Moreover, high-protein/high-meat diets may also be associated with increased risk for coronary heart disease due to intakes of saturated fat and cholesterol or even cancer [31]. Guidelines for diet should adhere closely to what has been clinically proved, and by this standard there is currently no basis to recommend high protein/high meat intake above the recommended dietary allowance for healthy adults [32–35]. Further investigation with large randomized controlled studies could provide more definitive evidence.

## Figures and Tables

**Table 1:** Disorders and health risks due to high protein/high meat intake (above 0.8 g protein/kg body weight/day) in adults.

ID	Subjects	Findings	References
Bone and calcium homeostasis			
1	11 healthy adults	Hypercalciuria	[8]
2	6 healthy adult males	(a) Hypercalciuria, (b) negative calcium balance	[9]
3	16 healthy adult females	(a) Hypercalciuria, (b) increased bone resorption	[10]
4	10 healthy adults	(a) Decreased estimated calcium balance, (b) increased risk for bone loss	[11]
5	85,900 adult females	Increased risk of forearm fracture	[12]
6	4 healthy adults and 4 patients with nephrolithiasis	(a) Hypercalciuria, (b) increased intestinal absorption of calcium	[13]
7	9 healthy adult males	Hypercalciuria	[14]
8	6 healthy adult males	Hypercalciuria	[15]
9	15 healthy adults	Hypercalciuria	[16]
10	6 healthy adult males	(a) Hypercalciuria, (b) the consumption of high calcium diets is unlikely to prevent the negative calcium balance and probable bone loss induced by the consumption of high protein diets	[17]
11	8 healthy adult males	Hypercalciuria	[18]
12	8 healthy adult males	Hypercalciuria	[19]
13	1035 adult females	A decrease in vegetable protein intake and an increase in animal protein intake increased bone loss and the risk of hip fracture	[20]

Renal function			
14	8 healthy adults with a history of renal stones	(a) Hyperuricosuria, (b) lower urine pH (c) Increased risk of forming crystals or stones in the urine	[21]
9	15 healthy adults	(a) Hyperuricosuria, (b) increased risk for uric acid stones	[16]
4	10 healthy adults	(a) Increased acid load to the kidney, (b) increased risk for stone formation	[11]
15	1624 adult females	Accelerated renal function decline in women with mild renal insufficiency	[22]
16	6 healthy adult males	Increased overall relative probability of forming stones	[23]
11	8 healthy adult males	(a) Hyperuricosuria, (b) decreased ability of urines to inhibit the agglomeration of calcium oxalate crystals	[18]
12	8 healthy adult males	(a) *Ι*ncreased glomerular filtration rate, (b) decreased fractional renal tubular reabsorption of calcium and urinary sodium	[19]

Cancer risk			
17	47,949 adult males	Elevated risk of colon cancer was associated with high intake of red meat	[24]
18	88,751 adult females	High intake of red meat increases the risk of colon cancer	[25]
19	18,139 adults	Meat intake positively associated with cancer risk (stomach, colon, rectal, pancreatic, bladder, breast, endometrial, and ovarian cancers)	[26]

Liver function			
20	2 healthy adult males on high protein supplements	(a) Elevations in transaminases, (b) hyperalbuminemia	[27]

Coronary blood flow			
21	36 adults	Precipitated progression of coronary artery disease through increases in lipid deposition and inflammatory and coagulation pathways	[28]
